# Predictors of brain infarction in adult patients on extracorporeal membrane oxygenation: an observational cohort study

**DOI:** 10.1038/s41598-021-83157-5

**Published:** 2021-02-15

**Authors:** Riccardo Iacobelli, Alexander Fletcher-Sandersjöö, Caroline Lindblad, Boris Keselman, Eric Peter Thelin, Lars Mikael Broman

**Affiliations:** 1 Department of Pediatric Perioperative Medicine and Intensive Care, ECMO Centre Karolinska, Astrid Lindgren Children’s Hospital, Karolinska University Hospital, 171 76 Stockholm, Sweden; 2 Department of Neurosurgery, Karolinska University Hospital, Stockholm, Sweden; 3 Department of Clinical Neuroscience, Karolinska Institutet, Stockholm, Sweden; 4 Department of Neurology, Karolinska University Hospital, Stockholm, Sweden; 5 Department of Physiology and Pharmacology, Karolinska Institutet, Stockholm, Sweden

**Keywords:** Stroke, Risk factors, Epidemiology, Circulation, Respiration

## Abstract

Non-hemorrhagic brain infarction (BI) is a recognized complication in adults treated with extracorporeal membrane oxygenation (ECMO) and associated with increased mortality. However, predictors of BI in these patients are poorly understood. The aim of this study was to identify predictors of BI in ECMO-treated adult patients. We conducted an observational cohort study of all adult patients treated with venovenous or venoarterial (VA) ECMO at our center between 2010 and 2018. The primary endpoint was a computed tomography (CT) verified BI. Logistic regression models were employed to identify BI predictors. In total, 275 patients were included, of whom 41 (15%) developed a BI. Pre-ECMO Simplified Acute Physiology Score III, pre-ECMO cardiac arrest, VA ECMO and conversion between ECMO modes were identified as predictors of BI. In the multivariable analysis, VA ECMO demonstrated independent risk association. VA ECMO also remained the independent BI predictor in a sub-group analysis excluding patients who did not undergo a head CT scan during ECMO treatment. The incidence of BI in adult ECMO patients may be higher than previously believed and is independently associated with VA ECMO mode. Larger prospective trials are warranted to validate these findings and ascertain their clinical significance.

## Introduction

Extracorporeal membrane oxygenation (ECMO) may be a life-saving treatment for patients with severe refractory lung and/or heart failure, when conventional intensive care fails. However, in addition to the critical condition of the patient, ECMO treatment itself is associated with significant morbidity and mortality^1^. Neurological complications are among the leading causes of death and disability in ECMO patients^1,2^. Of these, non-hemorrhagic brain infarction (BI) has a reported incidence of 1.7–8% and a particularly poor prognosis, with a 60–95% mortality rate^1,3–7^.

In the clinical setting, identifying risk factors for BI may help to identify infarctions early on or prevent them entirely. However, most studies investigating predictors of BI in ECMO patients have focused on pediatric and neonatal populations^2,3^, or combined ischemic stroke with intracranial hemorrhage (ICH) in their risk factor analyses^5,6,8^. To date, only two studies have analyzed predictors of BI in ECMO treated adults, identifying lactate > 10 mmol/L and on-admission platelet count > 350 × 10^9^/L as independent predictors of BI^4,7^. Results from registry studies also suggest that the incidence of BI may be higher in venoarterial (VA) ECMO patients compared to venovenous (VV) support, but this has yet to be confirmed in larger studies with common risk factor analysis^5,6^.

In this retrospective single-center cohort study, we aimed to identify predictors of BI in adults treated with ECMO.

## Material and methods

### Patients

All adult patients (≥ 18 years) who were treated with ECMO at our center between January 2010 and December 2018 were eligible for inclusion. Patients with BI or ICH on admission, and those partially treated at another ECMO center, were excluded. To reduce the influence of precipitating events, patients were also required to have been treated with ECMO for at least 12 h before decannulation or BI detection. The outcome variable was the presence of BI on a computed tomography (CT) scan during ECMO treatment. Absence of a head CT scan was deemed as “no BI”, although a sub-group analysis excluding patients who did not undergo a CT scan was performed as well. The study was performed in accordance with relevant guidelines and regulations and approved by the Stockholm Regional Ethical Review Board, who waived the need for informed consent (DNR: 2018-830/31).

### Patient management during ECMO treatment

In most cases, ECMO treatment was commenced at the referring hospital and the patient was then transported to our ECMO intensive care unit (ICU)^9,10^. Anticoagulation was achieved by an intravenous (i.v.) starting bolus of 50 IE/kg bodyweight followed by a continuous i.v. infusion of unfractionated heparin targeting an activated partial thromboplastin time (APTT) of 1.5–2 times the upper normal value, which was assessed at least three times daily. Within 2–3 days following arrival to our ICU, a tracheostomy was performed, after which sedation was reduced with the goal of keeping the patient awake during ECMO treatment. The bedside nurse and attending physician monitored the central nervous system through neurological examinations. This included response to verbal directives or pain, brainstem reflexes, eye opening and pupil examinations. A head CT scan was not performed per default on admission but always if the patient developed neurological symptoms (e.g. seizures, pupillary abnormalities, confusion, focal neurological deficits or decreased levels of consciousness). Additional head CT scans were also performed whenever patients were referred for thoracic or abdominal CT scans, even in the absence of a neurological indication.

### Variables

Data was collected from the hospital’s digital medical charts. Possible predicting variables were recorded until discharge or detection of a BI, which in turn was determined by the time of the diagnostic CT scan. Pre-cannulation data included age, sex, weight, smoking habit, age-adjusted Charlson Comorbidity Index (age points were subtracted)^11^, Simplified Acute Physiology Score III (SAPS-3)^12^, Sequential Organ Failure Assessment (SOFA)^13^, Glasgow Coma Scale (GCS) prior to sedation^14^, cardiopulmonary resuscitation during hospitalization and the last arterial blood gas before ECMO commencement. ECMO data included indication for treatment, modality (VA or VV), circuit change, conversion between modalities as well as the biochemistry, international normalized ratio (INR), platelet count, hemoglobin, fibrinogen and antithrombin. Complications that arose during treatment, including atrial fibrillation/flutter, extracranial thrombosis and bleeding (including ICH), were also noted. Follow-up data were 30-day and 6-month mortality.

### Statistical analysis

Shapiro-Wilks test was used to test for normality of distribution. Normally distributed continuous data are presented as mean (± SD), non-parametric continuous data as median (interquartile range) and categorical data as count (%). A univariate logistic regression analysis was conducted with BI (dichotomized) as the dependent variable. Variables that showed a trend towards significance (p < 0.1) in the univariate regression were added to a step-down multivariable logistic regression model to determine independent predictors of BI. In the step-down model, the least significant factor was sequentially eliminated until only significant variables remained. Two supplementary regression analyses were also performed on VV ECMO patients or those who did not undergo a CT scan, respectively. The laboratory parameters were plotted longitudinally and analyzed graphically. Listwise deletion was used to handle missing data. Relative risk (RR) was calculated according to Altman and Sheskin.

The model met the assumptions for logistic regression. No unusual or influential data outliers were identified. Absence of multicollinearity was verified through computation of the Variance Inflation Factors for each variable, where a value ≥ 2.5 was considered evidence of multicollinearity^15^. The Box-Tidwell method was used to evaluate the linear relationships between continuous independent variables and the logit transformed dependent variable^16^.

The significance level was set to p < 0.05. All analyses were conducted using SPSS (IBM Corp. IBM SPSS Statistics, Version 25.0, 2017. Armonk, NY: IBM Corp.). Scatterplots were created using the ggplot2 package in R (Wickham H. ggplot2: Elegant Graphics for Data Analysis, 2016. NY: Springer-Verlag).

## Results

**Figure 1:**
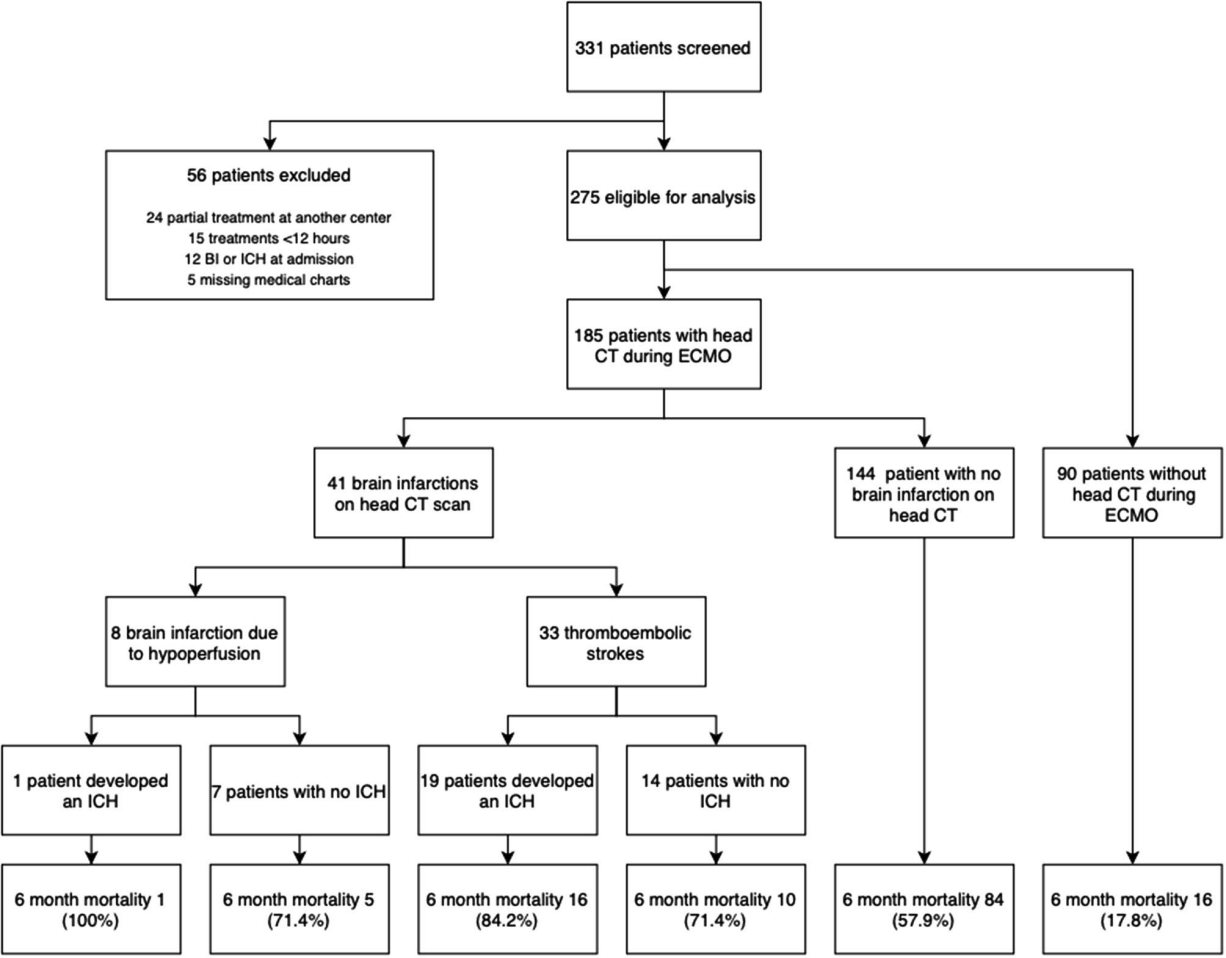
Schematic overview of patient inclusion and outcome. *BI* brain infarction, *ICH* intracerebral hemorrhage, *ECMO* extracorporeal membrane oxygenation.

During the study period, 331 adults (range 18–77 years), were admitted for ECMO treatment. Of these, 15 were excluded due to < 12 h of ECMO treatment, twelve were excluded due to the presence of acute stroke (BI or ICH) on admission, 24 were excluded because their ECMO treatment was partially conducted at a different hospital and five were excluded due to missing medical charts. Thus, 275 patients were included in the study (Fig. 1). The most common ECMO indication was sepsis syndromes (56%), and 139 patients were commenced on VA ECMO (50.5%), which was also the most common configuration at BI detection (61%) (Table 1).

**Table 1:** Univariate regression analysis predicting brain infarction during extracorporeal membrane oxygenation.

Variable	BI (n = 41)	No BI (n = 234)	p-value
**Pre-cannulation data**			
Age (years)	49 (38–58)	54 (35–62)	0.516
Male sex	26 (62%)	146 (62%)	0.952
Weight (kg)	82 (± 23), (2 missing, 5%)	83 (± 19), (7 missing, 3%)	0.859
Smoking	6 (14%)	42 (18%)	0.646
CCI	0.5 (0–2)	0 (0–1)	0.972
SAPS-3	84 (± 14), (8 missing, 19%)	78 (± 14), (48 missing, 21%)	**0.026**
GCS	13 (3–15), (8 missing, 19%)	13 (6–15), (48 missing, 21%)	0.090
SOFA total	14 (10–16), (11 missing, 26%)	13 (10–15), (60 missing, 26%)	0.286
SOFA coagulation	0 (0–2), (9 missing, 21%)	1 (0–2), (54 missing, 23%)	0.712
Cardiac arrest	14 (33%)	40 (17%)	**0.017**
**ABG analysis**			
pH	7.22 (7.09–7.33), (3 missing, 7%)	7.23 (7.14–7.31), (15 missing, 6%)	0.638
PaCO_2_ (kPa)	6.92 (5.34–8.99), (1 missing, 2%)	7.18 (5.9–9.2), (16 missing, 7%)	0.807
PaO_2_ (kPa)	7.45 (6.30–8.45), (3 missing, 7%)	7.60 (6.5–9.3), (16 missing, 7%)	0.155
p-lactate	4.00 (1.85–7.5), (5 missing, 12%)	2.75 (1.56–5.64), (23 missing, 10%)	0.113
**ECMO data**			
VA ECMO	30 (73%)	137 (50%)	**0.002**
Atrial fibrillation/flutter	12 (29%)	55 (24%)	0.482
Extracranial thrombosis	3 (7%)	21 (9%)	0.699
Extracranial bleeding	25 (60%)	124 (53%)	0.435
ECMO circuit change	12 (29%)	59 (25%)	0.647
Conversion of modality	12 (29%)	34 (15%)	**0.028**
Time until BI (days)	3 (2–6)	–	–

Normally distributed continuous data are presented as mean (± 1 SD), non-parametric continuous data as median (interquartile range) and categorical data as count (proportion).

Bold text in the p value column indicates a statistically significant correlation (p < 0.05).

*ABG* arterial blood gas, *BI* brain infarction, *CCI* Charlson comorbidity index, *ECMO* extracorporeal membrane oxygenation, *GCS* Glasgow Coma Scale, *SAPS-3* Simplified Acute Physiology Score III, *SOFA* Sequential Organ Failure Assessment, *VA* venoarterial.

### Brain infarction and patient outcome

**Figure 2:**
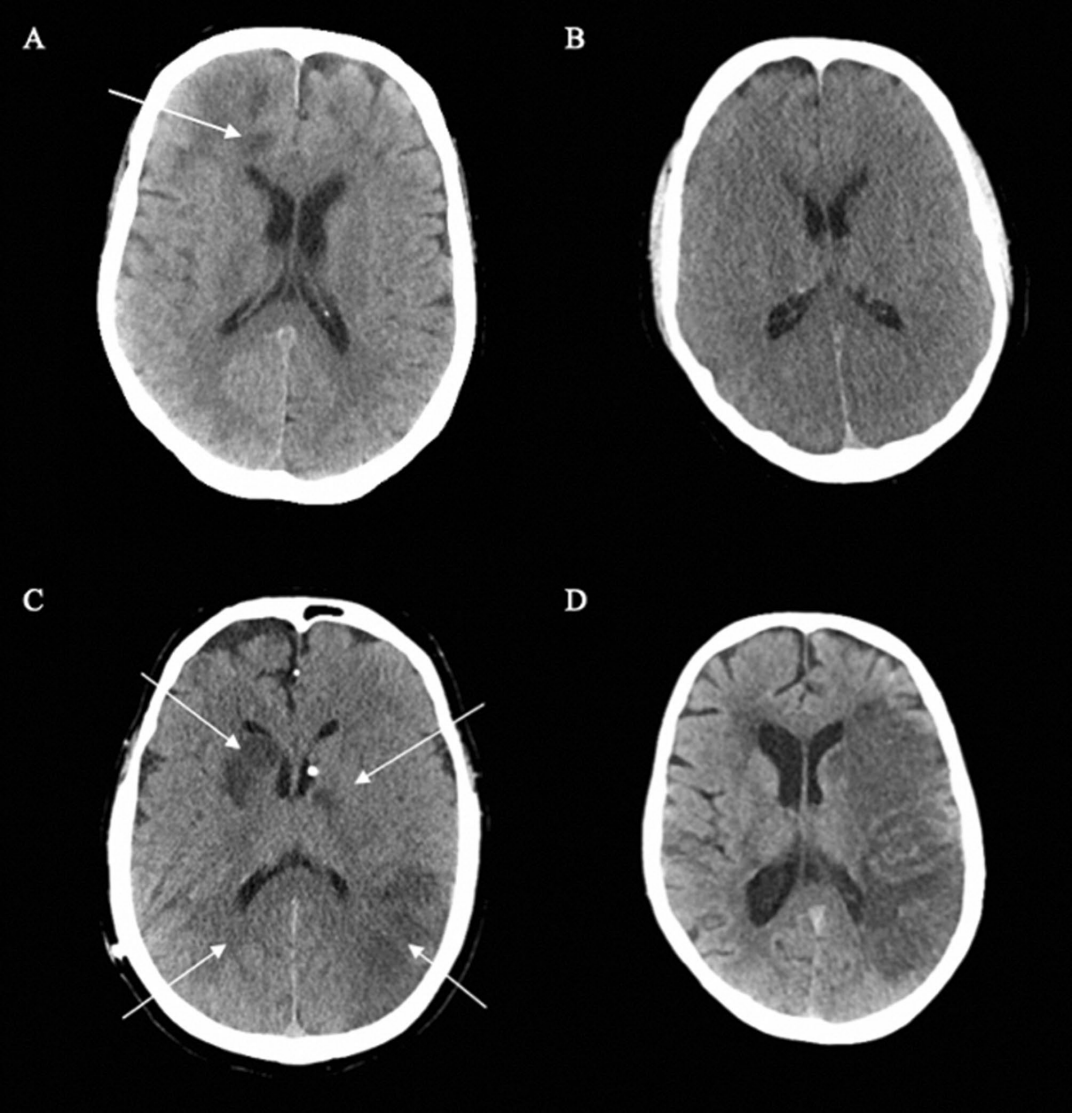
Four different examples of brain infarction during treatment on extracorporeal membrane oxygenation. Each arrow indicates a brain infarction that was diagnosed during ECMO treatment. (**A**) Watershed infarction; (**B**) hypoxic brain injury; (**C**) multiple ischemic cerebrovascular lesions; (**D**) middle cerebral artery occlusion.

**Figure 3:**
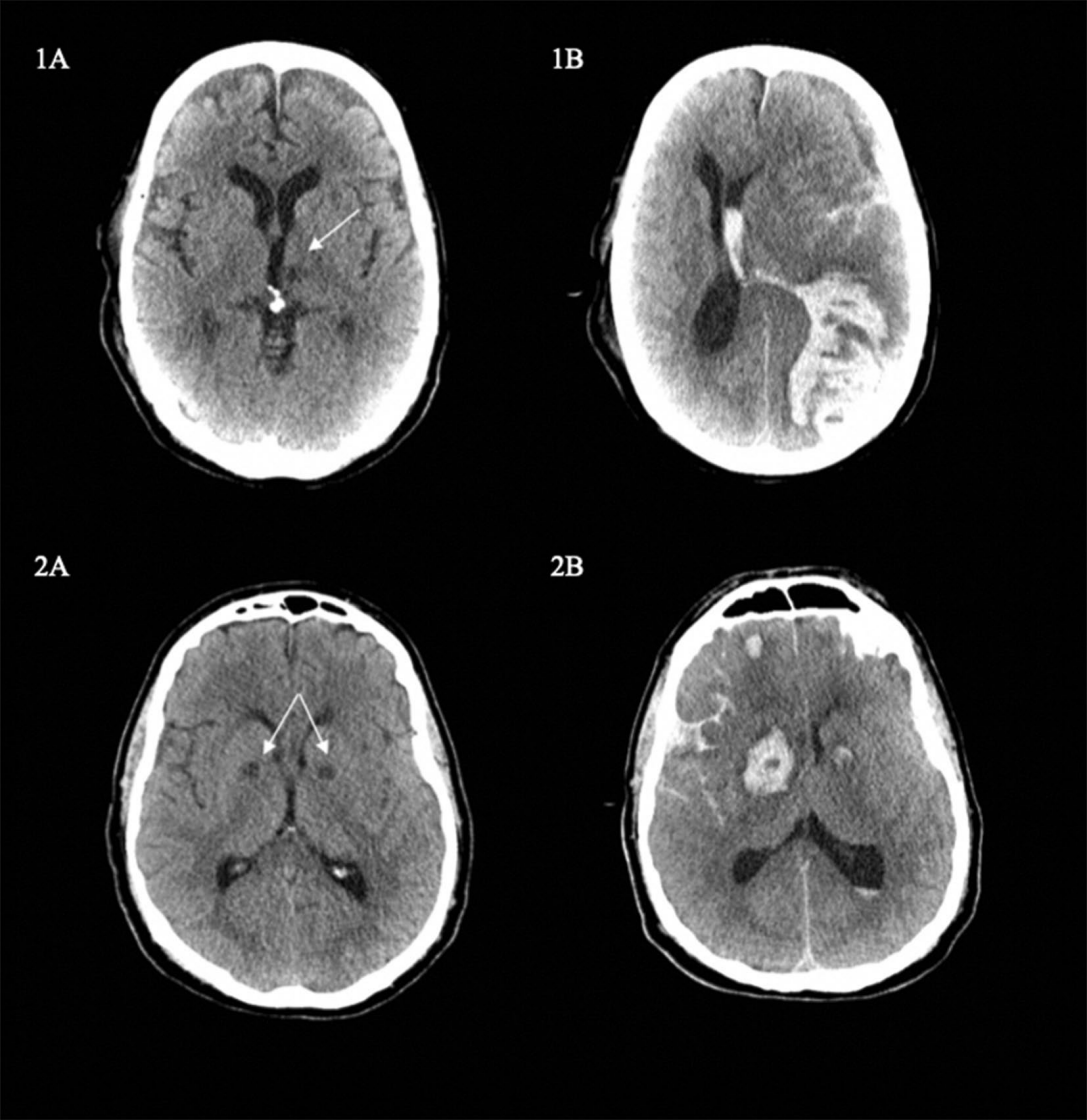
Two examples of hemorrhagic transformation following a brain infarction. Each arrow indicates a brain infarction that was diagnosed during ECMO treatment. Patient 1 had a radiologically verified brain infarction (**1A**) and developed neurological symptoms the next day following massive bleeding and herniation (**1B**). Patient 2 developed a symptomatic intracranial hemorrhage (**2B**) 3 days following bilateral brain infarction in the basal ganglia (**2A**).

In total, 41 patients (15%) were diagnosed with a BI during ECMO treatment. Of these, 33 lesions were deemed the result of focal ischemia due to thromboembolism while the remaining 8 were the result of cerebral hypoperfusion (Figs. 1, 2). Of note, 90 patients (33%) did not undergo a CT scan during ECMO treatment and were classified as not having developed a BI in the primary analysis. The median time from ECMO initiation to BI diagnosis was 3 days (IQR 2–6) (Table 1). Of the 41 patients diagnosed with a BI, 26 (63%) had symptoms that prompted the referral for a head CT scan. Compared to the non-BI cohort, patients who were diagnosed with a BI had a higher 30-day mortality (63% vs. 27%) and 6-month mortality (76% vs. 38%) (Fig. 1). Of note, 20 BIs (49%) underwent hemorrhagic transformation during ECMO treatment, meaning that BIs preceded 34% of all ICHs during the study period (Fig. 3).

### Predictors of brain infarction

In the univariate logistic regression model predicting BI development, increased risk of BI was associated with VA ECMO (p = 0.002), higher pre-ECMO SAPS-3 score (p = 0.026), pre-ECMO cardiac arrest (p = 0.017), and conversion between ECMO modalities (p = 0.028) (Table 1). Here, conversion from VV to VA ECMO carried a RR for BI development of 7.6 (CI 95% 2.6–23, p < 0.001), while the RR for conversion from VA to VV ECMO was 0.66 (CI 95% 0.2–2.5, p = 0.54). In the step-down multivariable analysis, VA ECMO showed sole independent risk association with BI development (p = 0.002, delta-R^2^ = 0.098, odds ratio (OR) = 4.86 (CI 95% 1.8–13)) (Table 2).

**Table 2:** Multivariable regression analysis predicting brain infarction during extracorporeal membrane oxygenation.

Variable	Univariate p-value	Nagelkerke’s R^2^	Multivariable p-value	OR (95% CI)
VA ECMO	**0.002**	0.098	**0.002**	4.86 (1.8–13.12)

Final results from the step-down multivariable logistic regression analysis that originally included the variables VA ECMO, SAPS-3, pre-ECMO cardiac arrest, GCS and conversion of modality.

Bold text in the p value column indicates a statistically significant correlation (p < 0.05).

*ECMO* extracorporeal membrane oxygenation, *GCS* Glasgow Coma Scale, *SAPS-3* Simplified Acute Physiology Score III, *VA* venoarterial.

**Figure 4:**
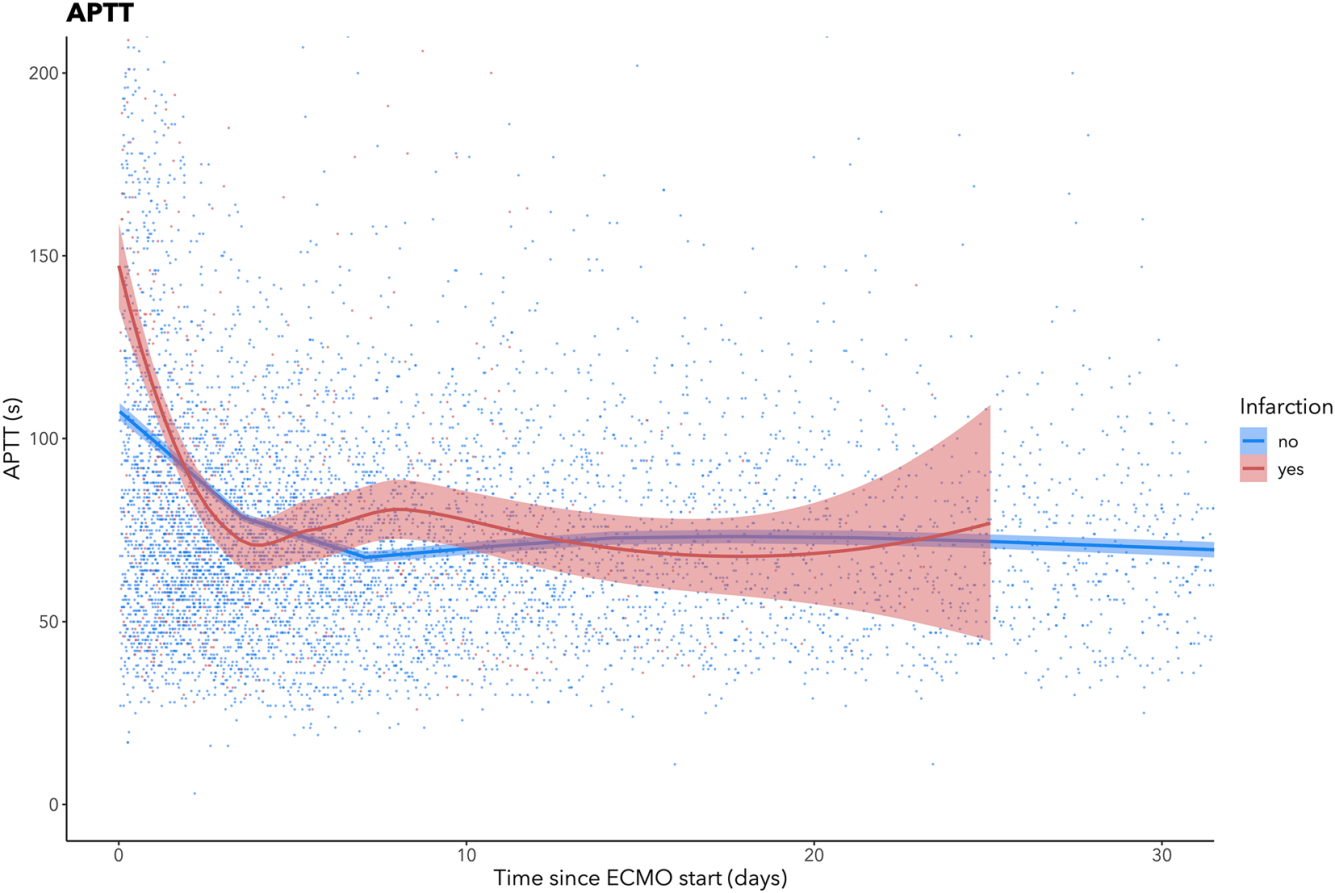
Scatterplot of activated partial thromboplastin time (APTT) depicted longitudinally and subdivided according to brain infarction status. Samples were collected during the first month of treatment or until BI detection. The red line represents patients that suffered brain infarction on ECMO, the blue line represents patients with no signs of infarction. The line indicates a LOWESS curve and the shaded area surrounding it indicates 95% confidence intervals. The graph was created using ggplot 2 (Wickham H. ggplot2: Elegant Graphics for Data Analysis, 2016. NY: Springer-Verlag).

Examined graphically, a trend was also seen in which patients who suffered from a BI had higher APTT on ECMO initiation (Fig. 4), as well as higher fibrinogen concentration, higher hemoglobin (Hb) concentration and decreased platelet count during the first week of treatment (Fig. 4, Supplementary Fig. 1).

### Sub-group analyses

In a sub-group analysis excluding patients who did not undergo a head CT scan during ECMO treatment (n = 90), VA ECMO was once again the sole independent BI predictor (p = 0.027) (Supplementary Tables 1 and Table 2). In an additional sub-group analysis of VV ECMO patients (n = 108), higher pre-ECMO serum lactate was identified as the only independent predictor of BI (p = 0.038) (Supplementary Table 3).

## Discussion

In this observational cohort study, we identified predictors of BI in ECMO treated adult patients. Out of 275 patients, 15% developed a BI. Pre-ECMO SAPS-3, pre-ECMO cardiac arrest, VA ECMO, and conversion between ECMO modes were identified as predictors of BI. VA ECMO was the only independent risk factor, and also remained significant when patients who did not perform a CT scan where excluded from the analysis. To the best of our knowledge, this is one of the largest studies on BI predictors in adult ECMO patients and contributes new data that could be important for patient management.

### Incidence and outcome

In total, 15% of our cohort were diagnosed with a BI during ECMO treatment. This is higher than the 1.7–8% incidence reported in previous studies^1,2,4–7^, and may be explained by our generous CT examination policy. About 37% of the lesions were diagnosed from a CT scan performed in the absence of neurological symptoms. Low utilization of neuroimaging as a contributor to underreporting of ECMO-associated intracranial pathologies has been suggested in several previous studies^3,4,17–19^. Thus, the incidence of BI in adult ECMO patients may be higher than previously estimated.

With regards to outcome, patients who developed BI had an increased 30-day and 6-month mortality. This is in line with results from previous studies^4,17,20^. In addition, 56% of the known deaths in these BI patients were accredited to the lesion or associated CNS complications. Naturally, the presence of an extensive BI may have led to withdrawal of ECMO as further treatment was considered futile. In summary, it appears that BI was associated with a higher mortality rate.

### Predictors of BI during ECMO treatment

We identified VA ECMO as an independent risk factor for BI, both in the primary analysis as well as in a sub-group analysis where we excluded patients who did not undergo a CT scan. Notably, a similar study by Omar et al. did not show any association between VA ECMO and an increased risk of BI^4^. However, a magnetic resonance imaging (MRI) study found that patients who had been treated with VA ECMO were more likely to display embolic cerebral infarctions and radiological signs of microembolization than their VV ECMO counterparts^21^. A long-term follow-up study by von Bahr and colleagues also found that patients previously treated with VA ECMO were more likely to display traces of past cerebrovascular lesions on MRI scans performed median 9 years (range 3–17) after ECMO treatment^22^. Furthermore, a study using transcranial ultrasound detected more microembolic signals in VA ECMO patients (81.8%), although they were also identified in the cerebral arteries of 26.2% of the included VV ECMO patients^23^. This latter finding may be of clinical importance in patients with a patent foramen ovale, where emboli from the ECMO circuit may reach the brain, particularly in those with elevated right heart pressures^24^. Conversely, one explanation behind the increased risk of BI using VA ECMO could be that blood is returned directly into the arteries without the lungs acting as a ‘filter’, thus allowing thrombi formed in the ECMO circuit to reach the cerebral circulation. This would primarily explain the embolic infarctions seen in our material, which was the most common cause of BI. The loss of a natural pulsatile flow seen in VA ECMO may also affect cerebral vessels, leading to hyper- or hypo-reactivity, which has been suggested to increase the risk for neurological injury^5,21^. Risk for differential hypoxemia on VA ECMO specifically may be an additional explanation^1^, where a high native cardiac output results in the hyper-oxygenated ECMO blood failing to reach the upper body’s circulation leaving the upper body end organs to be perfused with blood oxygenated by lungs with limited or no gas exchange. Depending on cannulation configuration and infusion site^25^, the brain may be then be subject to increased oxidative stress, cerebral vasoconstriction^1^, and thus increased risk for BI^24,26^. This might explain the patients in our cohort with a BI caused by hypoperfusion. Furthermore, in an effort to reduce selection bias, we also included the 40 patients who experienced a cardiac arrest before or during ECMO cannulation or those who underwent extracorporeal cardiopulmonary resuscitation (ECPR) (n = 14). However, only 14 ECPR patients met the inclusion criteria and although 36% (n = 5) developed a BI, the limited number of included patients limits any conclusion. However, we believe ECPR may be an important confounder and future studies may benefit from a separate analysis. Of the non-ECPR patients who experienced a cardiac arrest prior to ECMO treatment, nine (22%) suffered a BI. Other factors related to cardiogenic shock (for example low cerebral blood flow, hypoxia, acidosis, and hemostatic disorders due to liver failure) and reperfusion injury at ECMO initiation might also have precipitated BI^27^. In conclusion, VA ECMO was independently associated with an increased risk for BI development, which could be due to several risk factors associated with this type of modality. Of note, Nagelkerke’s pseudo-R^2^ for VA ECMO was 0.098 in our primary analysis. Although it may be viewed as positive that a single variable may explain almost 10% of CT verified BIs, it should also be viewed as an incentive for further studies on the subject, since there are probably additional predictors that impact the development of BI in adults treated with ECMO.

Conversion between ECMO modalities was also found to be a predictor of BI. This variable has not been assessed in previous studies. Thirty-two (70%) of the 46 conversions were from VV to VA ECMO, which carried a RR for BI development of 7.6 (as compared to an RR of 0.66 for conversion from VA to VV ECMO). Part of this association was probably due to VA ECMO mode itself, but may also be explained by the fact that the patient needed VA ECMO because of circulatory deterioration. Unpublished data from our unit has shown that the indications for > 95% of VV to VA conversions are right ventricular heart failure or circulatory shock, both of which have been identified as risk factors for ischemic stroke in non-ECMO cohorts^28^. Thus, patients subjected to conversion between ECMO modes had an increased risk of BI, but the causality behind the relationship remains unclear.

A high SAPS-3 score was associated with increased risk for BI development. This variable has not been assessed in previous studies of ECMO patients with neurological complications. The finding is to some degree expected, as the score is used to predict mortality for intensive care patients. We did not perform any subgroup analysis to identify whether there were any specific parameters of the SAPS-3 score that were associated with BI^12^, but these variables include age, malignancy and chronic heart failure, which have all been associated with an increased risk of stroke in the general population^28^. Thus, our results suggest that patients with high SAPS-3 prior to ECMO initiation were at increased risk of developing a BI during ECMO treatment, but the specific role of the underlying parameters needs to be further evaluated.

Lastly, we identified pre-ECMO cardiac arrest as a predictor of BI. It has previously been identified as significant risk factor for neurological complications in pediatric ECMO patients^29^. In our study, 75% of the global ischemic injuries (n = 6) were preceded by a pre-ECMO cardiac arrest. In a previous study of adults who developed BI on ECMO, a trend towards a significant association with pre-ECMO CPR was found^4^. This might be attributed to lesion development prior to ECMO initiation, but remains unclear as we did not perform routine CT scans upon admission and possible neurological symptoms prior to ECMO cannulation are difficult to evaluate in heavily sedated patients^30^.

### VV ECMO subgroup

While VA ECMO was independently associated with an increased risk of BI development, the complication occurred in 6.5% of the VV population as well. In this subgroup, BI was independently associated with high lactate levels prior to cannulation. This is in line with Omar et al., who found that lactate > 10 mmol/L was associated with ischemic stroke, although the study included both VV and VA ECMO patients^4^. This result may have been explained by hypoperfusion, however, only one of the BI in the VV ECMO subgroup had the radiological appearance of hypoperfusion. Another explanation may be that high lactate levels are seen in the acute phase of an ischemic stroke, which may indicate that these patients had an ongoing ischemia already upon ECMO initiation.

### Hemorrhagic transformation of BI

Patients who developed BI also had higher frequency of ICH compared to the non-BI cohort, with 49% of the infarcts undergoing hemorrhagic transformation—a well-known complication that contributes to increased mortality in ECMO patients^2^. In non-ECMO patients, Okada et al. reported transformation of embolic infarctions into ICH in almost 41% of their cases^31^. The management of anticoagulation in ECMO patients is less investigated in the treatment of embolic infarctions. However, of the 59 ICH identified in our study material, 20 (34%) had a verified BI before the bleeding event and, thus, a reperfusion injury was likely the cause. The conditions in ECMO may be similar to that of reperfusion in ischemic stroke patients, where ICH development is well-known risk^32^. It may be assumed that bleedings are associated with a high APTT and ischemic stroke with low APTT, however these findings indicate that both ICH and BI occur in the same patient category. Reducing APTT in order to reduce risk of ICH enlargement may, therefore, also be valid in subgroups of patients with BI to reduce the risk of hemorrhagic transformation^3^. In summary, ICH development was over-represented in patients with BI, which highlights the importance of anticoagulation management and warrants additional research in these patients.

### Laboratory hemostatic parameters

Examined graphically, a trend was seen in which patients who suffered from a BI had higher APTT at ECMO initiation (Fig. 4). The clinical relevance of this finding is unclear, as reduced APTT has been independently associated with ischemic stroke and subsequent neurological deterioration in non-ECMO patients^33^. Lower APTT has also been linked to a higher frequency of venous thromboembolism in ECMO patients^34^. One explanation may be over-correction and bolus effect of heparin on APTT after ECMO initiation, which would theoretically result in a prothrombotic tendency. It is also possible that a high APTT at ECMO initiation may be present in sicker patients due to more severe coagulopathy. However, as APTT is closely monitored and regulated in the ECMO ICU, it is difficult to draw any conclusion as to whether changes in APTT alone could predict a BI in this study. Fibrinogen concentration was also increased in BI-patients during the first week of ECMO treatment—which is the time when the majority of BI were diagnosed (Table 1).

### Limitations

This was a retrospective study, with its inherent limitations. Thirty-seven percent of the patients diagnosed with a BI did not show any neurological symptoms, and the clinical significance predicting these infarctions can be debated. Moreover, no routine CT scans were performed on admission which means that some patients may have developed a sub-clinical BI before ECMO treatment was started. We also chose to use data from both VA and VV ECMO patients in the primary analysis, as we believe this more accurately depicts the clinical setting of centers that offer both VA and VV ECMO and is especially important when conversions between modalities are considered.

### Clinical implications

Although this is a retrospective study, our research highlights the importance of closely monitoring predictors of BI. While ECMO is often a last resort treatment that is considered only after great scrutiny, it is important to identify the BI predictors that can be targeted with interventions or where earlier weaning from ECMO could be attempted. For example, patients with known risk factors for BI development should be subjected to more rigorous neurological monitoring, serial CT scanning and laboratory analysis of biomarkers of brain injury to identify BI as early as possible^35^. One example of such biomarker may be serial blood sampling of S100B, which our group has shown is able to detect BI in ECMO patients^36^. In this aspect, patients on VA ECMO should be prioritized. Guennec and colleagues recently reported on successful mechanical thrombectomy in two patients treated on VA ECMO, further highlighting the importance of diagnosing BI at an early stage^37^. Attempted earlier weaning from ECMO or conversion to VV ECMO when feasible may also be beneficial in high-risk patients. Considering the mortality associated with BI during ECMO, additional studies evaluating risk factors for BI in this patient group are warranted, as well as studies on management and predictors of outcome after the occurrence of a BI.

## Conclusions

Ischemic brain complications in adult ECMO patients may be more common than previously known and are independently associated with VA ECMO mode. Risk factor identification may help initiate steps to lower the risk of BI development or facilitate earlier diagnosis, and thereby improve outcome. Prospective trials are warranted to identify additional risk factors and their mechanisms of action.

## Supplementary Information

Supplementary Information.

## Data Availability

Data is available from the corresponding author upon request.
