# Supplementary material for: Predictors of brain infarction in adult patients on extracorporeal membrane oxygenation: an observational cohort study
Source: Sci Rep. 2021 Feb 15;11:3809. doi: 10.1038/s41598-021-83157-5 (PMC7884423; doi:10.1038/s41598-021-83157-5)
Supplement: Supplementary file 1 — Supplementary Information. [file 41598_2021_83157_MOESM1_ESM.docx]

Predictors of brain infarction in adult patients on extracorporeal membrane oxygenation: an observational cohort study

Riccardo Iacobelli* ^1^, Alexander Fletcher-Sandersjöö ^2,3^, Caroline Lindblad ^3^, Boris Keselman ^3,4^, Eric P Thelin ^3,4^, Lars Mikael Broman ^1,5^

^1^ ECMO Centre Karolinska, Department of Pediatric Perioperative Medicine and Intensive Care, Karolinska University Hospital, Stockholm, Sweden

^2^ Department of Neurosurgery, Karolinska University Hospital, Stockholm, Sweden

^3^ Department of Clinical Neuroscience, Karolinska Institutet, Stockholm, Sweden

^4^ Department of Neurovascular diseases, Karolinska University Hospital, Stockholm, Sweden

^5^ Department of Physiology and Pharmacology, Karolinska Institutet, Stockholm, Sweden.

Supplementary tables: 1-4

Supplementary figure: 1

**Corresponding author:**

Riccardo Iacobelli

+46 734041109

email: [riccardo.iacobelli@stud.ki.se](mailto:riccardo.iacobelli@stud.ki.se) / [riccardo.iacobelli@gmail.com](mailto:riccardo.iacobelli@gmail.com)

ORCID: 0000-0001-7536-3795

ECMO Centre Karolinska, Karolinska University Hospital, 171 76, Stockholm, Sweden

**Supplementary table 1. Univariate regression analysis predicting brain infarction during Extracorporeal membrane oxygenation.** Patients who did not undergo a CT scan during treatment have been excluded.

| **Variable** | **BI (n=41)** | **No BI (n=144)** | **p-value** |
| --- | --- | --- | --- |
| **Pre-cannulation data** |  |  |  |
| Age, years | 49 (38 – 58) | 55 (36 – 62) | 0.435 |
| Male sex | 26 (62%) | 94 (65%) | 0.612 |
| Weight (kg) | 82 (± 23), (2 missing, 5%) | 81 (± 18), (6 missing, 4%) | 0.763 |
| Smoker | 6 (14%) | 28 (19%) | 0.531 |
| CCI | 2 (0 – 3) | 2 (1 – 3) | 0.527 |
| SAPS-3 | 84 (± 14), (8 missing, 19%) | 80 (± 14), (28 missing, 19%) | 0.329 |
| GCS | 13 (4 – 15), (8 missing, 19%) | 13 (9 – 15), (29 missing, 20%) | 0.352 |
| SOFA total | 14 (10 – 16), (11 missing, 26%) | 14 (11 – 16), (36 missing, 25%) | 0.570 |
| SOFA coagulation | 0 (0 – 2), (9 missing, 21%) | 1 (0 – 2), (31 missing, 22%) | 0.445 |
| Cardiac arrest | 14 (33%) | 26 (18%) | 0.062 |
| pH | 7.22 (7.09 – 7.33), (3 missing, 7%) | 7.23 (7.14 – 7.32), (6 missing, 4%) | 0.878 |
| PaCO2 (kPa) | 6.92 (5.34 – 8.99), (1 missing, 2%) | 7.18 (5.9 – 9.18), (7 missing, 5%) | 0.594 |
| PaO2 (kPa) | 7.45 (6.30 – 8.45), (3 missing, 7%) | 7.55 (6.5 – 9.75), (6 missing, 4%) | 0.964 |
| p-lactate | 4.00 (1.85 – 7.5), (5 missing, 12%) | 3.4 (1.8 – 7.5), (13 missing, 9%) | 0.456 |
| **ECMO data** |  |  |  |
| VA ECMO | 35 (83%) | 77 (53%) | **0.027** |
| Atrial fibrillation/flutter | 12 (29%) | 44 (31%) | 0.874 |
| Extracranial thrombosis | 3 (7%) | 17 (12%) | 0.419 |
| Extracranial bleeding | 25 (60%) | 88 (61%) | 0.766 |
| ECMO circuit change | 12 (29%) | 49 (34%) | 0.568 |
| Conversion of modality | 12 (29%) | 28 (19%) | 0.309 |

Normally distributed continuous data are presented as mean (±1SD), non-parametric continuous data as median (interquartile range) and categorical data as count (proportion).

Bold text in the p value column indicates a statistically significant correlation (p < 0.05)

Abbreviations: ABG= Arterial Blood Gas; BI = Brain Infarction; CCI = Charlson comorbidity index; ECMO = extracorporeal membrane oxygenation; GCS = Glasgow Coma Scale; SAPS-3 = Simplified Acute Physiology Score III; SOFA = Sequential Organ Failure Assessment; VA = venoarterial

**Supplementary table 2: Multivariable regression analysis predicting brain infarction diagnosis during Extracorporeal membrane oxygenation (ECMO).** Final results from the step-down multivariable logistic regression analysis that included VA ECMO and pre-cannulation cardiac arrest. Patients who did not undergo a CT scan during ECMO treatment have been excluded.

| **Variable** | **Univariate p-value** | **﻿Nagelkerke's R^2^** | **Multivariable p-value** | **OR (95% CI)** |
| --- | --- | --- | --- | --- |
| VA ECMO | **0.027** | 0.043 | **0.027** | 2.37 (1.105 – 5.097) |

Bold text in the p value column indicates a statistically significant correlation (p < 0.05)

Abbreviations: VA = venoarterial

**Supplementary table 3. Univariate regression analysis predicting brain infarction during venovenous (VV) Extracorporeal membrane oxygenation.**

| **Variable** | **BI (n=7)** | **No BI (n=101)** | **p-value** |
| --- | --- | --- | --- |
| **Pre-cannulation data** |  |  |  |
| Age (years) | 55 (45 – 60) | 51 (35 – 62) | 0.680 |
| Male sex | 4 (57%) | 67 (66%) | 0.677 |
| Weight (kg) | 87 (± 39), (1 missing, 14%) | 80 (± 18), (2 missing, 2%) | 0.461 |
| Smoker | 1 (14%) | 20 (20%) | 0.669 |
| CCI | 2 (1.5 – 3) | 2 (0.5 – 3) | 0.445 |
| SAPS-3 | 75 (± 8.7), (2 missing, 29%) | 74 (± 12), (18 missing, 18%) | 0.822 |
| GCS | 13 (12 – 15), (2 missing, 29%) | 13 (12 – 15), (19 missing, 16%) | 0.792 |
| SOFA total | 9 (8 – 13), (2 missing, 29%) | 11 (9 – 14), (23 missing, 23%) | 0.499 |
| SOFA coagulation | 0 (0 – 0), (2 missing, 29%) | 0 (0 – 2), (18 missing, 18%) | 0.355 |
| Cardiac arrest | 1 (14%) | 5 (5%) | 0.322 |
| pH | 7.25 (7.09 – 7.305), (1 missing, 14%) | 7.28 (7.19 – 7.35), (6 missing, 6%) | 0.090 |
| PaCO2 (kPa) | 9.17 (7.83 – 11.65), (0 missing, 0%) | 7.16 (6.32 – 9), (6 missing, 6%) | 0.074 |
| PaO2 (kPa) | 7.64 (7.15 – 8.9), (0 missing, 0%) | 7.5 (6.46 – 8.65), (6 missing, 6%) | 0.145 |
| p-lactate | 4.3 (2.08 – 7.5), (1 missing, 14%) | 1.9 (1.25 – 2.9), (10 missing, 10%) | **0.012** |
| **ECMO data** |  |  |  |
| Atrial fibrillation/flutter | 1 (14%) | 21 (21%) | 0.682 |
| Extracranial thrombosis | 1 (14%) | 5 (5%) | 0.322 |
| Extracranial bleeding | 2 (29%) | 45 (45%) | 0.417 |
| ECMO circuit change | 1 (14%) | 16 (16%) | 0.913 |

Normally distributed continuous data are presented as mean (±1SD), non-parametric continuous data as median (interquartile range) and categorical data as count (proportion).

Bold text in the p value column indicates a statistically significant correlation (p < 0.05).

Abbreviations: ABG = Arterial Blood Gas; BI = Brain Infarction; CCI = Charlson comorbidity index; ECMO = extracorporeal membrane oxygenation; GCS = Glasgow Coma Scale; SAPS-3 = Simplified Acute Physiology Score III; SOFA = Sequential Organ Failure Assessment

**Supplementary table 4. Multivariable regression analysis predicting brain infarction during Extracorporeal membrane oxygenation (ECMO).** Final results from the step-down multivariable logistic regression analysis that included pre-cannulation serum pH, PaCO_2_ and lactate.

| **Variable** | **Univariate p-value** | **﻿Nagelkerke's R^2^** | **Multivariable p-value** | **OR (95% CI)** |
| --- | --- | --- | --- | --- |
| Pre-cannulation serum lactate | **0.012** | 0.116 | **0.038** | 1.451 (1.02 – 2.065) |

Bold text in the p value column indicates a statistically significant correlation (p < 0.05)

**Supplementary table 5. Patient characteristics sorted by ECMO modality.**

| **Variable** | **VA ECMO (n=167)** | **VV ECMO (n=108)** |
| --- | --- | --- |
| Age, years | 49 (35 – 62) | 48 (35 – 62) |
| Male sex | 100 (60%) | 71 (66%) |
| Weight (kg) | 83.6 (± 19) | 80.7 (± 19.8), |
| Smoking | 27 (16%) | 21 (19%) |
| CCI | 1.9 (± 1.8) | 1.9 (± 1.6) |
| SAPS-3 | 81.4 (± 14.3) | 74 (± 11.4) |
| GCS | 10.7 (± 4.8) | 12.4 (± 3.6) |
| SOFA total | 14.2 (± 3.6) | 11.4 (± 3.4) |
| SOFA coagulation | 1.4 (± 1.3) | 0.9 (± 1.1) |
| Pre-ECMO cardiac arrest | 47 (28%) | 6 (5.6%) |
| ABG analysis |  |  |
| pH | 7.18 (± 0.17) | 7.27 (± 0.12) |
| PaCO2 (kPa) | 8.4 (± 3.2) | 8 (± 2.5) |
| PaO2 (kPa) | 9 (± 8) | 8.5 (± 7) |
| p-lactate | 6 (± 5) | 2.55 (± 1.9) |
| Atrial fibrillation/flutter | 45 (27%) | 22 (20%) |
| Extracranial thrombosis | 18 (11%) | 6 (5.6%) |
| Extracranial bleeding | 101 (60%) | 47 (44%) |
| ECMO circuit change | 54 (32%) | 17 (16%) |
| **Brain infarction** | **34 (20.3%)** | **7 (6.5%)** |

Normally distributed continuous data are presented as mean (±1SD), non-parametric continuous data as median (interquartile range) and categorical data as count (proportion).

Abbreviations: ABG = Arterial Blood Gas; BI = Brain Infarction; CCI = Charlson comorbidity index; ECMO = extracorporeal membrane oxygenation; GCS = Glasgow Coma Scale; SAPS-3 = Simplified Acute Physiology Score III; SOFA = Sequential Organ Failure Assessment

|  |
| --- |
| **Supplementary figure 1.** Scatterplots of activated partial thromboplastin time (APTT), international normalized ratio (INR), platelets, hemoglobin, fibrinogen and antithrombin depicted longitudinally and subdivided according to brain infarction status. Samples were collected during the first month of treatment or until BI detection. The red line represents patients that suffered brain infarction on ECMO, the blue line represents patients with no signs of infarction. The smoothened line indicates a LOWESS curve and the shaded area surrounding it indicates 95% confidence intervals. |
